# Six-month incidence of hypertension and diabetes among adults with HIV in Tanzania: A prospective cohort study

**DOI:** 10.1371/journal.pgph.0001929

**Published:** 2023-08-21

**Authors:** Francis M. Sakita, Paige O’Leary, Sainikitha Prattipati, Monica S. Kessy, Kajiru G. Kilonzo, Blandina T. Mmbaga, Anzibert A. Rugakingira, Preeti Manavalan, Nathan M. Thielman, Dorothy Samuel, Julian T. Hertz

**Affiliations:** 1 Kilimanjaro Christian Medical Center, Moshi, Tanzania; 2 Department of Emergency Medicine, Duke University School of Medicine, Durham, North Carolina, United States of America; 3 Duke Global Health Institute, Duke University, Durham, North Carolina, United States of America; 4 Benjamin Mkapa Hospital, Dodoma, Tanzania; 5 Ministry of Health, Dodoma, Republic of Tanzania; 6 Department of Medicine, University of Florida College of Medicine, Gainesville, Florida, United States of America; PLOS: Public Library of Science, UNITED STATES

## Abstract

Data describing the incidence of hypertension and diabetes among people with HIV in sub-Saharan Africa remain sparse. In this study, adults with HIV were enrolled from a public clinic in Moshi, Tanzania (September 2020—March 2021). At enrollment, a survey was administered to collect information on comorbidities and medication use. Each participant’s blood pressure and point-of-care glucose were measured. Baseline hypertension was defined by blood pressure ≥140/90 mmHg or self-reported hypertension at enrollment. Baseline diabetes was defined by self-reported diabetes or hyperglycemia (fasting glucose ≥126 mg/dl or random glucose ≥200 mg/dl) at enrollment. At 6-month follow-up, participants’ blood pressure and point-of-care glucose were again measured. Incident hypertension was defined by self-report of new hypertension diagnosis or blood pressure ≥140/90 mmHg at follow-up in a participant without baseline hypertension. Incident diabetes was defined as self-report of new diabetes diagnosis or measured hyperglycemia at follow-up in a participant without baseline diabetes. During the study period, 477 participants were enrolled, of whom 310 did not have baseline hypertension and 457 did not have baseline diabetes. At six-month follow-up, 51 participants (95% CI: 38, 67) had new-onset hypertension, corresponding to an incidence of 33 new cases of hypertension per 100 person-years. Participants with incident hypertension at 6-month follow-up were more likely to have a history of alcohol use (90.2% vs. 73.7%, OR = 3.18, 95% CI:1.32–9.62, *p* = 0.008) and were older (mean age = 46.5 vs. 42.3, *p* = 0.027). At six-month follow-up, 8 participants (95% CI: 3, 16) had new-onset diabetes, corresponding to an incidence of 3 new cases of diabetes per 100 person-years. In conclusion, the incidence of elevated blood pressure and diabetes among Tanzanians with HIV is higher than what has been reported in high-income settings.

## Introduction

Sub-Saharan Africa (SSA) remains the epicenter of the HIV epidemic, accounting for 70% of the global burden of HIV [1, 2]. Within SSA, major advancements in HIV access to care have led to 11.8 million individuals now receiving antiretroviral therapy (ART) [2]. As people with HIV (PWH) receive treatment, they are living longer and developing chronic non-communicable diseases (NCDs), which is contributing to the demographic shift in SSA [3]. The population of elderly PWH is expected to grow substantially in future decades [2, 4], and the growing burden of age-related chronic NCDs such as hypertension and diabetes among PWH in SSA will place additional strain on local healthcare systems. As researchers and policymakers increasingly recognize the importance of addressing chronic non-communicable comorbidities among PWH in the region [5–7], more data are needed to describe the incidence of chronic NCD comorbidities to inform screening and treatment programs.

Recent studies from across SSA have examined the feasibility of leveraging established HIV care infrastructure to screen for and treat chronic NCD comorbidities among PLWH [8–11]. However, there is a lack of evidence-based care models to support large scale implementation of integrated care, resulting in limited NCD screening in most HIV care settings in the region [8]. In this context, cross-sectional studies from varied settings in SSA describe large proportions of PWH with hypertension and diabetes who were unaware of their NCD diagnosis and not taking anti-hypertensive or anti-hyperglycemic medications [9, 12]. In northern Tanzania, for example, the majority of PWH with hypertension or diabetes had never been screened for these diseases before, were unaware of their condition, and were not taking any NCD medications [13–15]. Routine NCD screening in HIV care settings could be an important first step in increasing awareness of NCDs among PWH and NCD comorbidities and, ultimately, improving NCD control among this population [15].

Despite the preliminary evidence that supports integrating NCD screening with HIV care [16], more data are needed about NCD incidence to inform integration plans and optimal screening intervals. Such data would inform policy decisions about the potential benefits of regular screening and optimal screening intervals. To address this gap, the purpose of this study was to determine the six-month incidence of hypertension and diabetes among PWH in northern Tanzania. A secondary aim was to identify correlates of incident hypertension among Tanzanians with HIV. Although there is a need for incidence data for a wide range of NCDs among PWH in SSA, we chose to focus on hypertension and diabetes in this study given preliminary evidence suggesting that the prevalence of undiagnosed hypertension and diabetes among PWH in Tanzania is large [13, 14, 17, 18].

## Methods

### Setting

Participants were enrolled from a government funded clinic at Majengo Care and Treatment Center (MCTC) in Moshi, Tanzania. MCTC provides approximately 1200 adults (approximately 900 women and 300 men) from Moshi and nearby rural areas with routine outpatient HIV care and ART. In northern Tanzania the prevalence of hypertension and diabetes among a random, community-based sample of adults was 28% and 6% in 2014, respectively [14, 19, 20].

### Participant selection

Between September 2020 until March 2021, patients over 18 years of age presenting to MCTC for routine HIV care were approached by local research assistants and offered enrollment. Patients were approached consecutively for enrollment while they were waiting for care at MCTC. There were no exclusion criteria, however patients were not eligible for re-enrollment if they had already been enrolled in the study during a prior visit to MCTC.

### Study procedures

All participants provided informed consent. Trained study staff administered surveys to collect information on comorbid medical conditions, medication use, and lifestyle behaviors. Each participant’s blood pressure, height, weight and glucose were measured at enrollment. Height was measured manually with the patient supine; weight was measured manually with a scale. The GlucoPlus Blood Glucose Monitoring System (GlucoPlus, Montreal, Canada) measured point-of-care glucose; the value was determined to be either fasting glucose if participants reported only having consumed water earlier in the day or random glucose if they reported having consumed any food or drink that day. The Beurer BM40 automatic blood pressure monitor (Beurer, Ulm, Germany) measured participant blood pressure according to international guidelines [21]: participants were in a seated position with both feet on the floor and arm at heart level, appropriately sized cuffs were used relative to the participant’s arm circumference, and a single blood pressure measurement was obtained after at least five minutes of rest. Lastly, staff collected HIV-related information, including length of ART therapy and most recent viral load, from patient medical records.

Participants followed-up with the research team during their routine 6-month visit to MCTC. At this time, research assistants measured participants’ blood pressure and point-of-care glucose again. All blood pressures at baseline and follow-up were measured on the same arm using the same sized cuff to maximize comparability.

### Study definitions

Hypertension was defined in accordance with World Health Organization guidelines for hypertension management [22]. Hypertension at baseline was either self-reported by participants, measured systolic blood pressure ≥140mmHg, or measured diastolic blood pressure ≥90mmHg. Diabetes was defined in accordance with American Diabetes Association guidelines [23]. Specifically, diabetes at baseline was defined by the following: participant self-report of diabetes; random glucose ≥200mg/dl if the participant reported consuming any food or drink earlier that day; or fasting glucose ≥126mg/dl if the participant reported consuming nothing other than water that day. Obesity was defined as measured body mass index (BMI) >30 kg/m^2^; calculated from participants weight and height measured at enrollment. Overweight was defined as BMI ≥25 kg/m2 and ≤30 kg/m^2^. Patients with recent HIV RNA viral load < 200 copies/ml were considered to be virologically suppressed. Participants with any self-reported formal secondary school education were considered to have secondary education. Those with any self-reported myocardial infarction (MI) or stroke in a first-degree relative were considered to have a family history of MI or stroke. Participants also self-reported lifestyle behaviors, including tobacco use, alcohol use, fruit and vegetable consumption and sedentary lifestyle, which was defined by the WHO guidelines as <150 minutes of moderately vigorous exercise per week [24].

Participants with baseline hypertension and baseline diabetes were excluded from incidence calculations. Incident hypertension was defined as measured systolic blood pressure ≥140mmHg at six-month follow-up, measured diastolic blood pressure ≥90mmHg at six-month follow-up, or self-report of new diagnosis of hypertension at six-month follow-up among participants without baseline hypertension. Incident diabetes was defined as random glucose ≥200 mg/dl at six-month follow-up, fasting glucose ≥126 mg/dl at six-month follow-up, or self-report of new diagnosis of diabetes at six-month follow-up among participants without baseline diabetes.

### Statistical analyses

The primary study outcome was the incidence of hypertension and diabetes among participants without baseline hypertension or diabetes (respectively) over a 6-month period. Patients lost to follow-up were excluded from the analysis. Ninety-five percent confidence intervals were constructed around point estimates for hypertension and diabetes incidence rates using the Poisson distribution. Univariate analyses were performed to compare characteristics among participants who developed hypertension and diabetes compared to participants who did not: Welch’s t-test was used for continuous variables, Pearson’s chi-squared was used for categorical variables, and the Fischer’s exact test was used when expected cell count < 5. A multivariate logistic regression, including variables with positive univariate association (p<0.10) with age and sex forced into the model, was performed to identify predictors of incident hypertension and diabetes among participants. The statistical analyses were performed using R suite. Sample size calculations for this study are reported elsewhere [13, 25, 26].

### Ethics

This study received ethics approval from the Tanzania National Institute for Medical Research and the institutional review board at Duke Health. This study had minimal level of risk and written informed consent was provided by all participants prior to enrollment. Authors did not have access to any information that could have identified participants during or after the study.

## Results

A total of 501 patients seeking routine care at MCTC were approached for study participation, of whom 500 (99.8%) consented and were enrolled in the study; of these, 477 participants completed six-month follow-up. Table 1 describes the participant sample (n = 310) without an initial self-reported diagnosis of hypertension or measured elevated blood pressure at baseline. The mean age (SD) of participants was 43.0 (10.5) and 88 (28.4%) were male. Most of these participants had completed primary education (n = 208, 67.1%), reported a history of alcohol use (n = 237, 76.5%), and led a sedentary lifestyle (n = 209, 67.4%). Most of the participants had a normal BMI (n = 175, 56.5%), and few participants reported a family history of cardiovascular disease (n = 53, 17.1%). The mean systolic blood pressure was 120.7 (SD = 10.8) and average diastolic blood pressure was 75.8 (SD = 7.5). On average, participants had been living with HIV for 5.6 (SD = 4.1) years and had been on ART for 5.0 (SD = 3.6) years.

**Table 1:** Participants without baseline hypertension or elevated blood pressure, Moshi, Tanzania (N = 310).

Participant Characteristics	Number of participants (%)
Male sex	88 (28.4%)
Age, mean (sd), years	43.0 (10.5)
Education	
None	20 (6.5%)
Primary	208 (67.1%)
Secondary	64 (20.6%)
Post-secondary	18 (5.8%)
Self-reported medical comorbidities	
Hypertension	0 (0%)
Diabetes	3 (1.0%)
Prior stroke	3 (1.0%)
Chronic kidney disease	1 (0.3%)
Hyperlipidemia	0 (0%)
History of tobacco use	77 (24.8%)
History of alcohol use	237 (76.5%)
Sedentary Lifestyle	209 (67.4%)
Body mass index	
Underweight (<18.5 kg/m^2^)	18 (5.8%)
Normal weight (18.5–25 kg/m^2^)	175 (56.5%)
Overweight (25–30 kg/m^2^)	72 (23.2%)
Obese (>30 kg/m^2^)	45 (14.5%)
Blood Pressure	
Systolic Blood Pressure, mean (sd), mmHg	120.7 (10.8)
Diastolic Blood Pressure, mean (sd),	75.8 (7.5)
Family history of cardiovascular disease	53 (17.1%)
HIV Duration, mean (sd), years	5.6 (4.1)
ART Duration, mean (sd), years	5.0 (3.6)
Current antiretroviral therapy	
TDF/lamivudine/dolutegravir	293 (94.5%)
TDF/lamivudine/efavirenz	5 (1.6%)
None	1 (0.3%)
Other	11 (3.6%)
Virologically suppressed (<200 copies/ml)	296 (95.5%)
CD4, mean (sd), count	483.9 (257.7)

At six-month follow-up, 51 participants (95% CI: 38, 67) without baseline hypertension met the study definition for incident hypertension, including 49 participants with measured elevated blood pressure at follow-up, 1 participant who reported an interim diagnosis of hypertension, and 1 participant with both measured elevated blood pressure and a self-reported interim diagnosis of hypertension. This results in an incidence of 33 new cases (95% CI: 25, 43) of hypertension per 100 person-years. Among the 51 participants with incident hypertension, the median (IQR) systolic blood pressure was 142 (138–149) mmHg and the median (IQR) diastolic blood pressure was 91 (84–96) mmHg.

Table 2 describes the participant population (n = 457) without an initial self-reported diagnosis of diabetes or hyperglycemia at baseline. The mean age (SD) of participants was 45.4 (11.1) and 121 (26.3%) were male. The majority of these participants had completed primary education (n = 325, 70.7%), reported a history of alcohol use (n = 360, 78.3%), and led a sedentary lifestyle (n = 316, 68.7%). On average, participants have been living with HIV for 5.6 (SD = 4.0) years and have been on ART therapy for 5.0 (SD = 3.6) years.

**Table 2:** Participants without baseline diabetes or hyperglycemia, Moshi, Tanzania (N = 457).

Patient Characteristics	Number of patients (%)
Male sex	120 (26.3%)
Age, mean (sd), years	45.3 (11.0)
Education	
None	29 (6.3%)
Primary	322 (70.5%)
Secondary	83 (18.2%)
Post-secondary	23 (5.0%)
Self-reported medical comorbidities	
Hypertension	50 (10.9%)
Diabetes	0 (0%)
Prior stroke	5 (1.1%)
Chronic kidney disease	2 (0.4%)
Hyperlipidemia	0 (0%)
History of tobacco use	109 (23.9%)
History of alcohol use	358 (78.3%)
Sedentary Lifestyle	314 (68.7%)
Body mass index	
Underweight (<18.5 kg/m^2^)	21 (4.6%)
Normal weight (18.5–25 kg/m^2^)	228 (49.9%)
Overweight (25–30 kg/m^2^)	117 (25.6%)
Obese (>30 kg/m^2^)	91 (19.9%)
Blood Pressure	
Systolic Blood Pressure, mean (sd), mmHg	131.3 (20.8)
Diastolic Blood Pressure, mean (sd), mmHg	81.5 (12.5)
Family history of cardiovascular disease	89 (19.5%)
HIV Duration, mean (sd), years	5.6 (4.0)
ART Duration, mean (sd), years	5.0 (3.6)
Current antiretroviral therapy	
TDF/lamivudine/dolutegravir	434 (95.0%)
TDF/lamivudine/efavirenz	9 (1.9%)
Other	13 (2.8%)
None	1 (0.2%)
Virologically suppressed (<200 copies/ml)	438 (95.8%)
CD4, mean (sd), count	487.7 (263.0)

At six-month follow-up, 8 participants (95% CI: 3, 16) without baseline diabetes or hyperglycemia at the initial visit developed incident diabetes, including 2 with measured hyperglycemia at follow-up, 3 with a self-reported interim diagnosis of diabetes, and 3 with both measured hyperglycemia and self-reported interim diagnosis of diabetes. This results in an incidence of 3 new cases (95% CI: 1, 6) of diabetes mellitus per 100 person-years.

Univariate associations between participant characteristics at baseline and incident hypertension are presented in Table 3. Participants with incident hypertension at 6-month follow-up were more likely to have a history of alcohol use (90.2% vs. 73.7%, OR = 3.18, 95% CI:1.32–9.62, *p* = 0.008) and were older (mean age = 46.5 vs. 42.3, *p* = 0.027) compared to participants without hypertension at the 6-month follow-up. No other participant characteristics were associated with incident hypertension, including HIV duration or ART duration.

**Table 3:** Associations between participant characteristics and incident hypertension (HTN) at the six-month follow-up visit in Moshi, Tanzania (N = 310).

Baseline Participant Characteristic	Participants without HTN at 6 months (N = 259)	Participants with HTN at 6 months (N = 51)	OR (95% CI)	p^a^
	N (%)	N (%)		
Male sex	72 (27.8%)	16 (31.4%)	1.19 (0.61, 2.26)	0.602
Secondary education	73 (28.2%)	9 (17.6%)	0.55 (0.24, 1.15)	0.117
Comorbidity of diabetes	1 (0.4%)	2 (3.9%)	9.79 (0.78, 311.16)	0.076
Hyperglycemia	6 (2.3%)	1 (2.0%)	0.94 (0.04, 5.88)	0.956
History of tobacco use	63 (24.3%)	14 (27.5%)	1.18 (0.58, 2.30)	0.631
History of alcohol use	191 (73.7%)	46 (90.2%)	3.18 (1.32, 9.62)	0.008*
Sedentary lifestyle	76 (29.3%)	19 (37.3%)	1.43 (0.75, 2.67)	0.270
Family history of cardiovascular disease	44 (17.0%)	9 (17.6%)	1.36 (0.29, 10.68)	0.717
	**Participants without HTN at 6 months (n = 259)**	**Participants with HTN at 6 months (N = 51)**		**p** ^**b**^
	**Mean (sd)**	**Mean (sd)**		
Age, years	42.3 (10.0)	46.5 (12.3)		0.027*
BMI, kg/m^2^	24.3 (4.7)	24.2 (4.8)		0.920
HIV duration, years	5.6 (4.1)	5.1 (3.9)		0.413
ART duration, years	5.1 (3.7)	4.6 (3.5)		0.413
CD4 count	483.3 (250.3)	487.0 (295.0)		0.413

^a^Pearson’s chi squared test

^b^Welch’s t-test

*statistically significant of *p* < 0.05

Univariate associations between participant characteristics and incident diabetes were not assessed since only eight participants developed incident diabetes during the study follow-up period.

## Discussion

This study is among the first to assess the 6-month incidence of elevated blood pressure and diabetes among PWH in Tanzania. Of participants without baseline hypertension, nearly 1 in 6 PWH developed elevated blood pressure in 6 months. Older participants and those with a history of alcohol use were more likely to develop elevated blood pressure compared to participants without hypertension. We also observed a handful of cases of incident diabetes.

In our cohort, we found the incidence of elevated blood pressure among PWH to be 33 (95% CI: 25–43) per 100 person-years, which is somewhat higher than what has been reported in other studies of hypertension incidence from SSA and high-income settings. Prior studies in Uganda, Ethiopia, South Africa, Ghana, and elsewhere in Tanzania reported the incidence of new hypertension among PWH to be between 5.4 to 32 per 100 person-years [27–30]. In general, the reported incidence of hypertension among PWH in SSA is higher than what has been reported in research from high-income settings: recent studies from Norway and the United States reported that the incidence of hypertension among PWH to be 3–6 cases per 100 person-years [31, 32]. In our setting, the high incidence of elevated blood pressure is paired with a lack of hypertension screening in routine HIV-related care [14], underscoring the need for routine hypertension screening among PWH in Tanzania. Participants’s age and alcohol use was significantly associated with incident elevated blood pressure, similar to other studies in SSA, highlighting the need to focus on addressing traditonally associated hypertension risk factors in this population [28].

In addition to traditional risk factors, factors specific to HIV may also contribute to hypertension in PWH. For example, there appears to be a clear association between treated HIV and hypertension compared to untreated HIV [33, 34]. Yet, whether this increased risk is due to the return to health phenomena associated with treatment or specific ART classes is less clear, and evidence invesigating the link between specific ART regimens and hypertension are mixed [35, 36]. Our study was not suited to evaluate associations between incident hypertension and ART (95% of our participants were on the same dolutegravir-based regimen–TLD), however some studies in SSA have found an association between older NRTI, NNRTI and PI agents (namely zidovudine, stavudine, nevirapine, and lopinavir-ritonavir) and hypertension [28, 29]. Moreover, the increasingly recognized associations between integrase inhibitors and obesity and other metabolic complications is of concern [37, 38], as these conditions tend to predispose invidiuals to hypertension and atherosclerotic cardiovascular (ASCVD) disease possibly increasing cardiovascular-related morbidity and mortality. As > 90% of all PWH in SSA are currently on an integrase based regimen (including participants in our sample), and as HIV is a known independent risk factor for ASCVD-related events, this may be problematic [39–42]. Further research investigating the associations between ART, hypertension and cardiovascular disease in PWH are needed.

In our cohort, we found the incidence of diabetes among PWH to be 3 (95% CI: 1–6) per 100 person-years, similar to the results of prior studies in SSA: a recent systematic review found that incident diabetes among PWH in SSA to be between 0.4 and 6 per 100 person-years [43]. In high-income settings such as the United States and France, the incidence of diabetes among PWH is generally lower (0.2–1.4 cases per 100 person-years) [44–47]. The higher incidence of diabetes observed in our study and others from SSA again underscores the need for greater attention to prevention, screening, and treatment of non-communicable comorbidities among the aging population of PWH in SSA.

This study had several limitations. First, we only recruited participants from those engaged in routine HIV care in Tanzania, so our findings might not be generalizable to those not engaged in HIV care, including those with undiagnosed HIV and those with untreated HIV. Secondly, our study might be underpowered in detecting all associations between participant characteristics and incident hypertension. Additionally, the 6 month follow up time period may not have been long enough to fully understand the correlates associated with incident hypertension or diabetes. Thirdly, we defined incident hypertension by a single blood pressure measurement at follow-up; multiple blood pressure measurements on multiple occasions would have provided a more accurate measure of incident hypertension, as recommended by international guidelines. Follow-up blood pressures were measured on only one occasion to accommodate the study timeline and the existing six-month follow-up system at MCTC, and reliance on a single blood pressure measurement may have resulted in over-estimation of incident hypertension. However, a recent study in Kenya found that the sensitivity and specifity of a single blood pressure measurement >140/90 mmHg for detecting hypertension was 80% and 84%, respectively, when compared to ambulatory blood pressure monitoring [48]. Assuming a single blood pressure measurement had similar diagnostic accuracy in our study population, the use of ambulatory blood pressure monitoring would likely have identified a similarly large number of incident hypertension cases. Additional longer-term studies with ambulatory blood pressure monitoring are needed to more accurately determine hypertension incidence in this population. Finally, we used random or fasting glucose levels to define incident diabetes, rather than hemoglobin A1c levels. Although hemoglobin A1c levels are generally preferred for defining diabetes mellitus, hemoglobin A1c is less reliable in diagnosing diabetes in PWH [49, 50].

In conclusion, we found a relatively high 6-month incidence of elevated blood pressure and diabetes among PWH in Tanzania. These findings call attention to the need for more robust NCD screening mechanisms among PWH in Tanzania and suggest that screening for hypertension and diabetes should be done frequently in this population. More research is needed to understand the mechanism of hypertension and diabetes risk among PWH in SSA and to develop interventions to target the burden of these NCDs and their associated risk factors in this population.

## Supporting information

S1 Checklist STROBE checklist. (DOCX)

S1 Data Full study dataset. (XLSX)

S1 Text Inclusivity in global research. (DOCX)
